# Professionally led support groups for people living with advanced or metastatic cancer: a systematic scoping review of effectiveness and factors critical to implementation success within real-world healthcare and community settings

**DOI:** 10.1007/s11764-023-01515-w

**Published:** 2024-01-08

**Authors:** Zhicheng Li, Kitty-Jean Laginha, Frances Boyle, Michele Daly, Fiona Dinner, Pia Hirsch, Kim Hobbs, Laura Kirsten, Carolyn Mazariego, Ros McAuley, Mary O’Brien, Amanda O’Reilly, Natalie Taylor, Lisa Tobin, Sophie Lewis, Andrea L Smith

**Affiliations:** 1 The Daffodil Centre, University of Sydney, a joint venture with Cancer Council NSW, Rm 111b, Edward Ford Building (A27), Camperdown, NSW 2006 Australia; 2 Faculty of Medicine and Health, University of Sydney, Sydney, NSW Australia; 3 Cancer Institute NSW, Consumer Advisory Panel, Sydney, NSW Australia; 4 Thursday Girls Group, Melbourne, Australia; 5 Advanced Breast Cancer Group, Brisbane, Qld Australia; 6 Westmead Centre for Gynaecological Cancers, Westmead, NSW Australia; 7 Nepean Cancer Care Centre, Penrith, NSW Australia; 8 Faculty of Medicine and Health, University of New South Wales, Sydney, NSW Australia; 9 Melbourne, Australia; 10 Sydney, NSW Australia; 11 Breast Cancer Network Australia, Camberwell, VIC Australia

**Keywords:** Advanced cancer, Metastatic cancer, Support groups, Peer support, Psychosocial support, Metastatic survivorship

## Abstract

**Purpose:**

To examine the effectiveness of professionally led support groups for people with advanced or metastatic cancer, and identify factors critical to implementation success within real-world settings.

**Methods:**

Databases (MEDLINE; PsychINFO; CINAHL) and grey literature were searched for empirical publications and evaluations. Articles were screened for eligibility and data systematically extracted, charted and summarised using a modified scoping review methodology. Implementation factors were mapped using Proctor’s implementation framework and the Consolidated Framework for Implementation Research 2.0.

**Results:**

A total of 1691 publications were identified; 19 were eligible for inclusion (8 randomised controlled trials, 7 qualitative studies, 2 cohort studies, 2 mixed methods studies). Most (*n*=18) studies focused on tumour-specific support groups. Evidence supported professionally led support groups in reducing mood disturbances (*n*=5), distress (i.e. traumatic stress, depression) (*n*=4) and pain (*n*=2). Other benefits included social connectedness (*n*=6), addressing existential distress (*n*=5), information and knowledge (*n*=6), empowerment and sense of control (*n*=2), relationships with families (*n*=2) and communication with health professionals (*n*=2). Thirteen studies identified factors predicting successful adoption, implementation or sustainment, including acceptability (*n*=12; 63%), feasibility (*n*=6; 32%) and appropriateness (*n*=1; 5%).

Key determinants of successful implementation included group leaders’ skills/experience, mode of operation, travelling distance, group composition and membership and resourcing.

**Conclusions:**

Professionally led tumour-specific support groups demonstrate effectiveness in reducing mood disturbances, distress and pain among patients. Successful implementation hinges on factors such as leadership expertise, operational methods and resource allocation.

**Implications for Cancer Survivors:**

Professionally led support groups may fill an important gap in supportive care for people with advanced or metastatic cancer.

**Supplementary Information:**

The online version contains supplementary material available at 10.1007/s11764-023-01515-w.

## Introduction

Historically, survival after a diagnosis of advanced or metastatic cancer (a solid or haematological malignancy unlikely to be cured with treatment) has been poor. However, improvements in treatment, such as targeted therapies, immunotherapies and antibody-drug conjugates, are driving the emergence of a growing population of patients living long-term with cancers that are treatable but unlikely to be curable [1–5]. For these patients, the aim of treatment is to slow progression of the cancer, prolong life and control the symptoms [1, 2]. Given these recent advancements in treatment, a priority for many patients is now maintaining quality of life.

Supportive care in cancer includes the prevention and management of the symptoms and side effects of cancer and its treatment from diagnosis to end-of-life and includes support for patients, their families and their caregivers [6]. Comprehensive supportive care can reportedly improve patients’ quality of life by helping them manage the complex physical, psychosocial and practical challenges that accompany living with metastatic cancer [7–10]. However, many cancer supportive care and survivorship services may be relatively unaware of the growing population of people living long term with advanced or metastatic cancer and their unique and often complex supportive care needs [11, 12]. Consequently, many people with advanced or metastatic cancer report feeling isolated and report high levels of unmet supportive care needs [7, 13–15]. The most prevalent of these are unmet health system, informational, psychological, physical and daily living needs [7].

Support groups for people with advanced or metastatic cancer are a relatively low-cost, effective and readily implementable way of addressing these unmet needs [16]. Support groups are an established part of cancer supportive care, playing a critical role in educating patients about their cancer, empowering them to take control of their care, improving their confidence in interactions with healthcare professionals and ultimately driving changes in health-seeking and health-promoting behaviours [17–19]. Cancer support groups have also been shown to improve the psychosocial wellbeing and overall quality of life of people affected by cancer [20].

Although the support group model of care has been widely adopted around the world by cancer service delivery organisations, relatively few offer groups specifically for people living with advanced or metastatic cancer. The need for specialised or stage-specific cancer support groups was highlighted by research reporting on the experiences of women with metastatic breast cancer of stage-specific versus mixed-stage online breast cancer support groups based in the USA. In contrast to those participating in stage-specific breast cancer groups, those participating in mixed-stage groups reported feeling stigmatised, marginalised and silenced [21]. In Australia, the need for specialised advanced or metastatic cancer support groups has long been recognised [22], as have the challenges of meeting the needs of those with advanced or metastatic cancer in mixed-stage groups where the majority of patients/participants are being treated with curative intent for a pre-defined period of time [17, 21, 23, 24]. The culture of many cancer support groups is strongly influenced by the dominant ‘recovery narrative’ that emphasises the positive aspects of being a cancer survivor and the importance of adopting an optimistic outlook to beat cancer, promote recovery, prevent recurrence and adjust to life beyond cancer [25]. Such groups can be challenging for those living with metastatic cancer who are dealing with the complexity of ongoing and frequently changing treatment regimens, alongside, of course, living with the knowledge of incurability, and coping with an uncertain future [23]. Despite the widespread recognition of the need for specialised advanced or metastatic support groups, relatively few exist, even in countries such as Australia where the need has been acknowledged. This is possibly due to the additional complexity, cost and risks associated with running such groups [26].

Support groups may be peer-led (facilitated by someone with lived experience of cancer) or professionally led (facilitated by healthcare professionals such as psychologists, counsellors, social workers or oncology nurses) [27]. Regardless of who leads the group, the social support element of groups is based on principles of peer-based mutual aid, self-help and empowerment. It is widely accepted that people who face similar disease-related issues can empower one another through regular, close social contact and support [28–30]. Professionally led support groups may also incorporate therapeutic interventions such as behavioural adaptations and cognitive skills or draw on particular psychotherapeutic models of care such as cognitive behavioural therapy (CBT) or supportive-expressive group therapy (SEGT) [20, 31, 32]. CBT is a psychotherapeutic approach that emphasises how a person’s thoughts and behaviours affect the way they feel [33]. SEGT is designed to encourage participants with life-threatening illnesses to express their emotions, thoughts and concerns about their illness and its effect on their lives [34].

Support group leaders play a critical role in determining the success or failure of the group [35, 36]. Success requires the support group leader to possess a complex mix of knowledge, skills and attributes [37, 38]. Common challenges faced by support group leaders include member recruitment, dealing with participants’ disease progression, maintaining boundaries and leader fatigue or burnout [36, 38, 39]. In addition, group leaders may need to take on significant administrative responsibilities, sometimes with little support or funding [40]. Consequently, more than half of group leaders report experiencing various difficulties [35]. In the early or curative setting, many groups are led by a peer support group leader who has completed active treatment. This is not possible in the metastatic or advanced cancer setting as treatment is typically life-long. Peers who do lead metastatic or advanced groups therefore have the additional challenge of balancing the running of the group while managing their own health [41].

Given the complexity of running a support group and the added challenges of peer-led support groups in the metastatic and advanced cancer setting [42, 43], we sought to understand the evidence concerning professionally led support groups for people with advanced or metastatic cancer. Several reviews have investigated professionally led [20, 44–46] or peer-led [30] cancer support groups in general. A 2013 Cochrane review evaluated psychological interventions for women with metastatic breast cancer but limited inclusion to RCTs [47]. We were unable to identify any reviews that focused on professionally led support groups for people with advanced or metastatic cancer. Additionally, of the reviews we could identify, few reported on challenges relating to implementing and sustaining stage-specific groups for people with advanced or metastatic cancer.

To address this research gap, this scoping review aimed to map, synthesise and report on the evidence relating to professionally led support groups for people diagnosed with advanced or metastatic cancer or their family members/carers. In developing this review, two key areas of interest were identified as the focus for data extraction and synthesis: (1) what is the nature of the evidence relating to the effectiveness of professionally led support groups for people with advanced or metastatic cancer? and (2) what is the nature of the evidence relating to factors that support or hinder the implementation of these groups in real-world healthcare and community settings? Given the growing number of people living with advanced or metastatic cancer, the extensive evidence base supporting use of support groups in cancer, and the aforementioned challenges of peer leadership, we believed that this review would provide a timely and important contribution to the literature.

## Method

### Study design

Scoping reviews use rigorous and transparent methods to comprehensively identify and analyse the literature pertaining to a research question. They are suitable for the current research as the evidence base concerning professionally led support groups for advanced or metastatic cancer is complex and heterogeneous. Methods draw on Arksey and O’Malley’s original scoping review framework and subsequent extensions, primarily the 2020 updated Joanna Briggs Institute (JBI) methodological guidance for scoping reviews [48–52]. Reporting is in accordance with the Preferred Reporting Items for Systematic Reviews and Meta-Analyses extension for Scoping Reviews (PRISMA-ScR) [53]. As this review provides an overview of evidence regardless of methodological quality or risk of bias, no quality assessment was conducted, consistent with PRISMA-ScR guidelines.

### Population, concept and context

The population included people attending, running or supporting the implementation of support groups for people affected by advanced or metastatic cancer. For the purposes of this review, we used White and colleagues’ criteria for treatable but not curable cancers. These criteria identify cancers that are highly unlikely to be eradicated and that, in the absence of other more imminent causes of death, are likely to lead to death [1]. The concept was support groups, defined as an ongoing gathering of individuals who share common experiences [54]. For the purpose of this review, support groups can take place in person, online or via teleconference but must include the giving and receiving of emotional and practical support as well as ongoing, real-time interaction between group members [54]. Online forums and social media pages that did not involve ongoing, real-time interaction between members were not considered to be support groups and were excluded. The context included all service settings (e.g. hospital and community) in any geographical location.

### Research question, data sources and search strategy

The research question was “What has been reported about professionally led support groups for people affected by advanced or metastatic cancer?” A search strategy was developed in consultation with an academic librarian and adapted for each database (Supplementary file S1). MEDLINE and PsycINFO were searched using the Ovid platform; CINAHL was searched using the EBSCO host platform. Searches were run on 15 December 2021. In addition to electronic databases, websites targeting organisations involved in the delivery of support groups for advanced or metastatic cancer care were searched to identify additional documents (e.g. reports and evaluations).

### Study selection and data extraction

The titles and abstracts of all unique records were independently screened by two reviewers to generate a list of potentially eligible articles. The full-text articles were retrieved and independently assessed against the selection criteria (Table 1) by two reviewers. Any disagreements were resolved through discussion until consensus was reached, with a third reviewer consulted as necessary. A data extraction form was developed and tested using a subset of five studies. Minor adaptations to the form were made with input from the team during the data extraction process. Two researchers independently extracted the data which were then verified by a third researcher. Data extracted included study characteristics (i.e. author, year of publication, study country, study design); population (i.e. cancer type and stage); intervention (i.e. mode of delivery, frequency, facilitator); and outcomes. Outcome data included (a) intervention effectiveness and (b) factors affecting implementation. Preliminary review of the studies indicated that a 1989 US study of women with metastatic breast cancer reported a survival advantage associated with attending professionally led SEGT-informed support groups [55]. Survival was therefore initially considered a possible outcome; however, given this survival advantage was not replicated by five subsequent studies [56–60], survival was excluded as an outcome of interest.

**Table 1:** Scoping review inclusion and exclusion criteria

Domain	Inclusion criterion	Exclusion criterion
Publication type	Peer-reviewed empirical studies Evaluations/reports identified in grey literature	Reviews, editorials, commentaries, letters to the editor, dissertations, study protocols and conference abstracts
Population characteristics	People attending, running or supporting the implementation of support groups for people affected by advanced or metastatic cancer (NB: We used White and colleagues criteria to determine which cancers could be classified as advanced or metastatic [1]) Support groups must be for people ≥ 18 years of age	Incurable chronic diseases that are not cancer (e.g. motor neuron disease, Huntington’s disease); < 18 years of age
Country	Any geographical location	None
Intervention	Professionally led cancer support groups that: • are ongoing (or intended to continue if shown to be effective) • include the giving and receiving of emotional or practical support • take place in person, online or via teleconference • include ongoing, real-time interaction between group members • led by at least one trained professional such as healthcare professional, social worker or counsellor	Peer-led support groups that are led by someone with a cancer diagnosis or a family member of someone with cancer Short-term (6 sessions or less) group therapy programs or interventions to support self-management of symptoms, treatment, side effects (e.g. pain management, antiemetic prophylaxis), anxiety etc. Online forums and social media pages that did not involve ongoing, real-time interaction between members.
Data	Reporting data relevant to the effectiveness or implementation of the intervention	Not reporting data relevant to the effectiveness or implementation of the intervention
Language	Studies published in English	Studies published in other languages

As few of the studies had an explicit implementation focus, we systematically searched each article for data or information that we could retrospectively link to implementation, including information reported in the methods and discussion. Factors that we believed were potentially related to implementation were mapped to the domains and constructs of the Consolidated Framework for Implementation Research (CFIR) 2.0 and then linked to Proctor’s Implementation Outcomes Framework [61, 62]. CFIR is a meta-theoretical determinant framework that provides a menu of constructs operating at the level of the individual, innovation, organisation or wider environment that have been associated with effective implementation. The CFIR constructs were drawn from 19 frameworks or related theories, including seminal works such as Rogers’ Diffusion of Innovations Theory and Greenhalgh and colleagues’ Diffusion of Innovations in Service Organisations [63, 64]. CFIR can also be used to guide the tailoring of implementation strategies and adaptations for the innovation being implemented. As recommended by Reilly et al. (and subsequently adopted by Damschroder et al. in their CFIR Outcomes Addendum), we differentiated between implementation antecedents and implementation outcomes [61, 65, 66]. According to Reilly et al., implementation antecedents are the factors that predict dissemination or implementation. Under this guidance, acceptability, appropriateness and feasibility do not match constitutive definitions of dissemination or implementation but rather reflect theoretical antecedents of implementation outcomes.

## Results

**Fig. 1:**
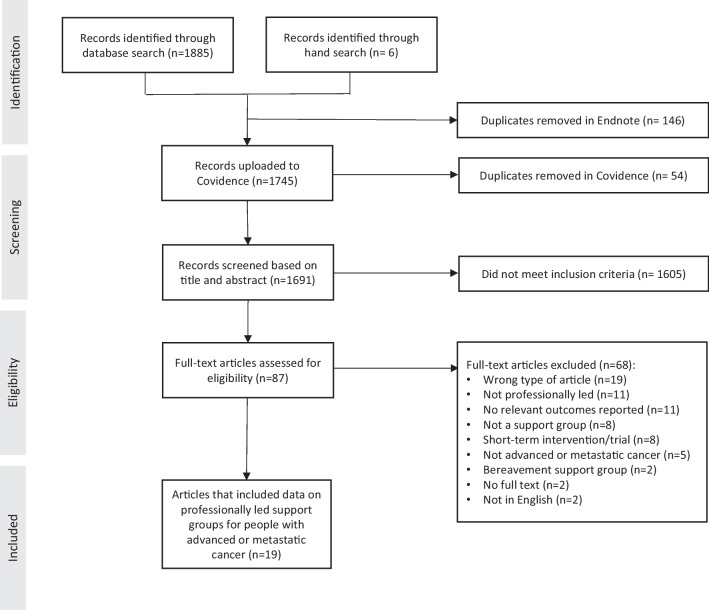
PRISMA diagram

In total, 1691 unique publications were identified. After screening abstracts, full-text articles and reports (*n*=87), 19 studies were eligible for inclusion (Fig. 1).

### Study characteristics

Characteristics of all included studies are summarised in Table 2. All 19 studies were conducted in high-income, Western countries (USA: *n*=6; Canada: *n*=7; Australia: *n*=4; UK: *n*=2). Eight were randomised controlled trials (RCTs), 7 were qualitative studies, 2 were cohort studies and 2 were mixed methods studies. There were 1841 participants in total, including 1571 patients (across 19 studies), 262 caregivers or family members (across 4 studies) and 8 healthcare professionals (across 2 studies). Sample sizes ranged from 8 to 238 participants, with an average of 97 participants per study. The average sample size per study for the RCTs and non-RCT studies were 141 and 59 per study, respectively.

**Table 2:** Studies reporting data on professionally led advanced or metastatic cancer support groups (n = 19)

Author; country (year)	Study aim/objective	Participants	Study design (follow-up for RCTs)	Facilitators	Intervention components
Spiegel et al.; USA (1981) [67]	To systematically evaluate the effects of weekly supportive group meetings	*N* = 58 women with MBC; *n*_intervention_ = 34, *n*_control_ = 24	RCT (1 year)	A psychiatrist or social worker and a counsellor who had breast cancer in remission	Weekly psychological group meetings (90 min) with or without a self-hypnosis exercise for pain
Spiegel and Bloom; USA (1983) [68]	To study the effect of group therapy on pain and mood disturbance	Same as Spiegel et al. (1981)	RCT (1 year)	Same as Spiegel et al. (1981)	Same as Spiegel et al. (1981)
Edmonds et al.; Canada (1999) [69]	To assess the effects of a psychological intervention on the mood and quality of life and changes in other psychological measures	*N* = 66 women with MBC; *n*_intervention_ = 30, *n*_control_ = 36	RCT (1.2 year)	A principal therapist (clinical psychologist) and a co-therapist: a doctoral candidate in psychology or a social worker	Weekly meetings (120 min) for 35 weeks and a 20-week course of cognitive behavioural assignments
Classen et al.; USA (2001) [70]	To evaluate the effectiveness of SEGT for reducing mood disturbance and traumatic stress symptoms in women with MBC	*N* = 125 women with MBC; *n*_intervention_ = 64, *n*_control_ = 61	RCT (1 year)	Two therapists including a psychiatrist/psychologist and social worker	Weekly therapy sessions (90 min) with facilitated discussion of prearranged themes
Goodwin et al.; Canada (2001) [59]	To determine whether the addition of SEGT to routine care influenced the survival, psychological functioning, experience of pain and quality of life	*N* = 235 women with MBC; *n*_intervention_ = 158, *n*_control_ = 77	RCT (1 year)	Two leaders including psychiatrists, psychologists, social workers and nurses	Weekly meetings of SEGT (90 min)
Bordeleau, et al.; Canada (2003) [71]	To evaluate the effect of SEGT on health-related quality of life	Same as Goodwin et al. (2001)	RCT (1 year)	Same as Goodwin et al. (2001)	Same as Goodwin et al. (2001)
Lemieux et al.; Canada (2006) [60]	To examine the impact of SEGT on health care costs	*N* = 125 women with MBC (a subset of the 235 patients included in the trial); *n*_intervention_ = 82, *n*_control_ = 43	RCT (1 year)	Same as Goodwin et al. (2001)	Same as Goodwin et al. (2001)
Kissane et al.; Australia (2007) [58]	To assess the impact of SEGT on survival, psychosocial outcomes and treatment adherence	*N* = 227 women with MBC; *n*_intervention_ = 147, *n*_control_ = 80	RCT (2 years)	Two therapists, one with medical/oncological background and one with group/psychotherapy background	Weekly SEGT (90 min) and 60-min relaxation class every 4 months
Feldman; USA (1993) [72]	To evaluate an innovative, non-traditional, multi-disciplinary group approach to support patients with prostate cancer through their course of treatment	*N* = 37, including 21 patients with metastatic prostate cancer and 16 family members	Cohort study	A clinical social worker and a therapeutic recreation specialist	Weekly meetings (90 min) on prearranged subjects of interest and scheduled trips
O’Brien et al.; Australia (2008) [73]	To assess the clinical effectiveness of a professionally led hybrid face-to-face and telephone support group for women with MBC in urban and rural areas	*N* = 21 women with MBC (including 6 women from rural and 6 from regional areas)	Cohort study	Two female therapists (psychiatrist and social worker; occupational therapist and social worker)	Weekly SEGT meetings (60 min) of a hybrid format—concurrent face-to-face and telephone/teleconference
Spiegel and Yalom; USA (1978) [31]	To describe the structure and outcomes of a support group program for MBC	Sample size not specified, over 40 women with MBC attended the program	Qualitative (Observation)	Psychiatrists/psychiatric residents, a social worker, counsellors	Weekly meetings (90 min) with occasional training of self-hypnosis or meditation
Spiegel and Glafkides; USA (1983) [74]	To understand the content of support groups and study the effect of patients’ direct confrontation with the physical deterioration and death of fellow MBC patients. It is part of a RCT (Spiegel, Bloom & Yalom 1981)	*N* = 11 women with MBC who were actively involved in one of the intervention groups	Qualitative (Observation)	A psychiatrist and a counsellor who had breast cancer in remission	Weekly meetings (90 min) guided by SEGT and a self-hypnosis exercise for pain
Kissane et al.; Australia (2004) [75]	To qualitatively review the application of SEGT in a RCT (Kissane et al. 2007), including its structure and framework, therapists and factors impacting its success	*N* = 147 women with MBC who were randomised to the intervention group of the RCT	Qualitative (Observation)	Same as Kissane et al. (2007)	Same as Kissane et al. (2007)
Leadbeater; UK (2004) [76]	To evaluate the support group for women with MBC in meeting their needs and identifying benefits and difficulties in attending	*N* = 8 women with MBC	Qualitative (Survey of open-ended questions)	A social worker who was also trained as a counsellor	SEGT meetings (frequency and duration of the meetings were not reported)
Bell et al.; Canada (2010) [77]	To study cancer support groups to generate insights into what makes them attractive and how they might be best structured and composed	*N* = 25 women with metastatic cancer, 23 participated in the in-depth interview^a^	Qualitative (interviews and observation)	Professionally facilitated; no further details reported	Fortnightly group meetings, including a meditation period followed by open sharing among members guided by the facilitator
Walker et al.; Canada (2010) [78]	To assess the application of SEGT to women with advanced ovarian cancer by exploring both positive and negative experiences and how SEGT affected patients’ relationships with medical professional	Study 1: *N* = 8 patients with advanced ovarian cancer Study 2: *N* = 9, including 6 patients with advanced ovarian cancer and 3 oncology professionals	Qualitative (Interviews)	Two registered clinical psychologists, one of each gender	Weekly meetings of SEGT
Thursday Girl’s Group; Australia (2020) [79]	To evaluate the Thursday Girls Group, a support group for women with MBC and their partners (two separate groups)	*N* = 20; including 14 women with MBC and 6 partners of women with MBC	Qualitative (Interviews and focus groups)	Two social workers (voluntary) with experience in palliative care	Weekly meetings (90 min) based on SEGT with activities and informal socialisation outside the meetings
Moore et al.; UK (2008) [43]	To evaluate the process of establishing a support group for people affected by mesothelioma	*N* = 11, including 6 support group participants (4 patients with mesothelioma and 2 family members) and 5 facilitators	Mixed methods (Survey of open and closed questions)	Oncology healthcare professionals (a patient information officer, a psychologist, a psychotherapist and two lung cancer nurses)	Monthly face-to-face meetings, some with speakers
Kanter et al.; Canada (2014) [80]	To identify characteristics of brain tumour group participants in relation to attendance frequency and compare themes of discussion in patient and caregiver groups	*N* = 137 patients with brain tumours *N* = 238 caregivers Qualitative analysis was conducted on 79 patient sessions and 76 caregiver sessions	Mixed methods (Review of medical charts and content analysis of support group topics)	Two neuro-oncology health professionals from nursing, social work or occupational therapy	Monthly meetings (90 min) with separate concurrent groups for patients and for caregivers

*MBC* metastatic breast cancer, *SEGT* supportive expressive group therapy

^a^The study reported on three support groups, only the metastatic cancer group met the inclusion criteria of the current review

Only one study reported on a mixed-tumour group [77]. The remaining 18 studies reported on tumour-specific support groups. Among them, 15 pertained to metastatic breast cancer, 1 to metastatic prostate cancer [72], 1 to advanced ovarian cancer [78], 1 to brain cancer [80] and 1 to mesothelioma [43]. Most of the studies had patient-only support groups (*n*=15). Two studies included groups specifically for family members and/or caregivers [79, 80] and two had groups that were open to patients, family members and/or caregivers [43, 72]. All support groups were delivered exclusively face-to-face, except for one that employed a hybrid mode of delivery, comprising simultaneous face-to-face and teleconference meetings [73]. All of the group leaders were trained professionals. The groups were typically facilitated by two leaders with different professional backgrounds such as psychiatry, psychology, counselling, oncology, allied health or social work. One study had a facilitator who had breast cancer in remission [67] and another had a patient representative to co-facilitate the group initially but this practice was discontinued due to the turnover of members and their medical situations [72]. Fifteen of the studies featured support groups that met weekly, one met fortnightly [77], two met monthly [43, 80] and no information was available for one study [76]. Meeting duration ranged from 1 to 2 h, during which group members were guided by the facilitators to discuss prearranged or spontaneous themes and share their experiences and emotions. Of the 19 studies, 15 reported support groups that drew on a psycho-theoretical framework: 10 were based on SEGT [58–60, 70, 71, 73, 75, 76, 78, 79]; 4 were based on group psychotherapy (primarily Yalom’s group psychotherapy) [31, 67, 68, 74]; one was based on group psychotherapy and CBT [69]. It was noted that some groups were based on SEGT initially, but they evolved over time to meet the needs of the individual members and of the group as a whole.

### Key findings

The effectiveness outcomes and perceived benefits of professionally led support groups for people affected by advanced or metastatic cancer are reported in Table 3.

**Table 3:** Effectiveness outcomes and perceived benefits of professionally led support groups for people affected by advanced or metastatic cancer

Outcomes	Number of studies	Measures/data collection methods	Key findings
Quantitative			
Mood	5	POMS, ABS	2 RCTs reported significant improvement [67, 70], 1 RCT reported partially significant improvement [59], 1 RCT reported no significant improvement [69], 1 cohort study reported significant improvement [73]
Distress			
Traumatic	3	IES	2 RCTs reported significant reduction in traumatic stress [58, 70],
stress			1 cohort study reported no significant reduction in traumatic stress [73]
Depression	1	MILP	1 RCT reported significant effect in preventing depression [58]
Quality of life	3	EORTC QLQ-C30, FLIC	Of the 3 RCTs reporting on quality of life, none reported significant effect on overall quality of life [58, 69, 71]
Pain	2	Pain rating scale developed by Spiegel and Bloom (1983)	2 RCTs reported significant improvement in self-reported pain [59, 68]
Qualitative and mixed methods			
Social connectedness	6	Observation, facilitators’ notes, interviews with participants and facilitators, survey	Connecting with other people with advanced or metastatic cancer, perceived support, sense of belonging, feeling less isolated, feeling understood [31, 43, 74, 76, 78, 79]
Existential distress	5	Observation, facilitators’ notes, interviews with participants and facilitators	Facing death/dying, acceptance of illness, meaning of life, fear and concerns about disease progression [31, 74, 77–79]
Information and knowledge	6	Observation, facilitators’ notes, interviews with participants and facilitators, survey	Sharing information about medical treatment, cancer diagnosis, available resources [43, 74, 77–80]
Empowerment and sense of control	2	Interviews with participants	Providing hope, improved perception of control and inner strength [78, 79]
Relationships	2	Observation	Improving relationships with families [31, 74]
Communication	2	Interviews with participants	Improving communication with health professionals [78, 79]

*ABS* Derogatis Affects Balance Scale, *FLIC* Functional Living Index for Cancer, *IEORTC* European Organization for Research and Treatment of Cancer, *IES* Impact of Event Scale, *MILP* Monash Interview for Liaison Psychiatry, *POMS* Profile of Mood States, *QLQ-C30* Quality of Life Questionnaire-Core 30

### Effectiveness outcomes

The quantitatively measured outcomes were consolidated into mood (reported in *n*=5 studies), distress (incorporating traumatic stress and depression, *n*=4), quality of life (*n*=3) and pain (*n*=2). Effectiveness outcomes that were reported by fewer than two quantitative studies are not reported in this review. These outcomes included cost-effectiveness, maladaptive coping responses, phobia, social support and repression.

Mood was measured in four RCTs using the Profile of Mood States (POMS) and one cohort study using the Derogatis Affects Balance Scale (ABS) [81]. POMS is a self-administered questionnaire consisting of six subscales on anxiety, depression, anger, vigour, fatigue and confusion, with the total score indicating general mood disturbance [82]. Classen and colleagues [70] and Spiegel and colleagues [67] reported significant improvement in reducing mood disturbances in the support group participants compared with control participants. Goodwin and colleagues [59] reported a significant interaction of intervention-group assignment with baseline POMS scores, suggesting those who had higher POMS scores (indicating more mood disturbances) at baseline benefited from the support group intervention, whereas those who had lower baseline POMS scores did not. Edmonds and colleagues [69] did not find any significant improvement in POMS scores between support group and control participants. In a cohort study using the ABS, a significant reduction in negative affect and an increase in positive affect (excluding the vigour subscale) were observed among the support group participants over a 12-month period [73].

Traumatic stress in response to cancer diagnosis was assessed in two RCTs and a cohort study using the Impact of Event Scale, a self-report measure for the occurrence of symptoms as a result of a stressful event [83]. Both trials reported significant declines in traumatic stress symptoms among the support group participants [58, 70]. A similar trend was observed in O’Brien and colleagues’ evaluation [73]; however, the reduction in stress symptoms over time was not significant. Depression was assessed in one RCT [58]. The authors reported that women with metastatic cancer who participated in the support group were less likely to develop depression compared to the control participants, measured using the Monash Interview for Liaison Psychiatry.

Quality of life was measured in three RCTs using the European Organization for Research and Treatment of Cancer (EORTC) Quality of Life Questionnaire-Core 30 (QLQ-C30) and the Functional Living Index for Cancer [58, 69, 71]. None reported significant effects of the intervention on overall quality of life. One trial showed a significant improvement among the support group participants in the social functioning domain of the EORTC QLQ-C30 [58].

The impact of support groups on reducing the experience of self-reported pain and suffering in women with metastatic breast cancer was reported in two RCTs [59, 68]. In the study conducted by Spiegel and Bloom [68], one of the intervention groups included self-hypnosis training for managing cancer-related pain in addition to group therapy sessions. Compared to the control group, the support group participants reported significantly less pain sensation and suffering, especially for those who participated in the additional self-hypnosis exercises. In a later study by Goodwin and colleagues [59], both intervention and control participants reported an increase in pain over the course of the study, but the support group participants reported less worsening of pain than did the control group participants. There was also a significant interaction of treatment-group assignment with baseline pain rating, suggesting those who had more pain at the outset benefited from the intervention, whereas those with lower baseline ratings did not.

### Perceived benefits

Seven qualitative and two mixed method studies reported on the benefits of attending professionally led support groups, drawing on data collected via surveys (*n*=2) of patients with metastatic or advanced cancer, interviews/focus groups (*n*=2) with support group participants and leaders, observation of the group meetings (*n*=3), content analysis of support group topics (*n*=1) and a combination of interviews and observation (*n*=1). Perceived benefits were grouped into the following thematic categories: (1) social connectedness (including connecting with other people with advanced or metastatic cancer, perceived support, sense of belonging, feeling less isolated and feeling understood); (2) existential distress (including facing death/dying, acceptance of illness, meaning of life, fear and concerns about disease progression); (3) information and knowledge (including information on medical treatment, cancer diagnosis and available resources); (4) empowerment and sense of control; (5) relationships with families; and (6) communication with health professionals (see Table 3).

One of the most frequently reported benefits was social connectedness (*n*=6) [31, 43, 74, 76, 78, 79]. Participating in a support group helped people with advanced or metastatic cancer and their partners connect with others in similar situations, foster a sense of belonging and acceptance and feel supported and less alone. For instance, among the questionnaire responses collected by Leadbeater in an evaluation of a support group for women with metastatic breast cancer, many members said that they had never met anyone with metastatic breast cancer prior to attending the group [76]. Being part of the group, thus, made them feel less alone [76]. In a group for partners of women with metastatic breast cancer, the group helped members open up about feelings and thoughts they felt they were unable to share with their partners [79].

Another benefit of professionally led support group pertained to gaining knowledge and information, reported in six studies [43, 74, 77–80]. For example, Kanter and colleagues found that both patients and carers used the groups to exchange and seek information about the disease and treatment [80].

Five studies reported that dealing with existential distress was an important benefit associated with attending support groups [31, 74, 77–79]. Coping with and facing end-of-life was a theme discussed actively and incidentally within groups. Although it was noted that this could be seen as ‘confronting’ or ‘distressing’ by some, especially those who were newly diagnosed with advanced or metastatic cancer [77], in general participants reported that groups were helpful in addressing the existential distress often experienced by this population through accepting their diagnosis and prognosis, adapting to the illness and normalising death and dying. For example, in their observations, Spiegel and Yalom found the support group to be beneficial in helping its members face death “realistically without denial but also without morbid rumination” (p. 244) and find meaning in the remainder of their lives [31].

Other perceived benefits included empowerment and regaining a sense of control (*n*=2) [78, 79], improving relationships with families (*n*=2) [31, 74] and improving communication with health professionals (*n*=2) [78, 79].

### Barriers and enablers to implementation and implementation strategies

Factors influencing the implementation of the support groups were identified in 13 studies (68%). Table 4 provides details of the barriers and enablers grouped according to CFIR domains and constructs and how they relate to the predictors of implementation success (implementation antecedents). The data were categorised as related to the acceptability (*n*=12; 63%), feasibility (*n*=6; 32%) and appropriateness (*n*=1; 5%) of support groups.

**Table 4:** Factors influencing the adoption, implementation and sustainment of professionally led support groups for people affected by advanced or metastatic cancer and potential strategies to address these factors

Factors	Implementation antecedent^a^	Implementation outcome^a^	Reported as a barrier	Reported as an enabler	Potential implementation strategies identified in the review
INNOVATION					
Adaptability					
Ability for group to adapt/evolve in organic and dynamic way, such as allowing for:	Feasibility	Sustainment		X	The ability of members to have input into content/programming of groups [72]
- The group model to change (e.g. move from a psychotherapeutic to mutual aid model) [79] - The group to move towards a more democratic mode (e.g. members’ ability to influence content of meetings/agenda) [72, 79] - The group to expand from ‘core’ meeting to peripheral activities under the control of members [31, 75, 79]				X	
Finding the right ‘fit’ (i.e. finding balance between ‘fidelity’ to a protocol and adaptability to needs of the particular group) [75]	Appropriateness	Adoption	X		
Complexity					
Maintaining adequate membership to keep group viable given members experience changing health status and some die [58, 75, 79]	Feasibility	Sustainment	X		Self-referral: a group’s ‘open-door policy’ (i.e. no referral needed) [79] Advertising and promotion of group (e.g. word of mouth—hospital staff and patients; flyers) [72]
INNER SETTING					
Culture: Recipient-centredness					
Reluctance from clinicians to refer or recommend due to perception groups would be unsettling to participants [31, 78, 79]	Feasibility	Adoption/implementation/sustainment	X		
Available resources: Funding					
Adequate resources (e.g. staff, facilities, consumables) - Ability of hospital to absorb group running costs [43]	Feasibility	Sustainment		X	
Lack of external funding/reliance on fundraising [79]	Feasibility	Sustainment	X		
Relational connections					
Collaboration between healthcare professionals from different disciplines and patients to set up and run group [43]	Feasibility	Adoption/implementation		X	
INDIVIDUALS					
Recipients: Needs					
The need to have a group that an individual can identify with, e.g. commonalities based on cancer type, role (carer versus patient), age, gender, dependent children [80]	Acceptability	Implementation/sustainment	X	X	Opportunities for members to interact in smaller groups beyond the formal group meetings, e.g. out-of-session add-ons/spin-offs, such as lunches, catch-ups, online social media groups [31, 75, 79]
Appropriate group composition or membership - Maintaining appropriate group size (6–12 members) [31, 43, 73, 75]	Acceptability	Sustainment		X	Continuity of membership (and facilitators) [79] Encouraging regular attendance by members supported group cohesion and sustainability [73, 79]
The need to travel to attend the group [58, 59, 76, 79]	Acceptability	Implementation	X		Option of joining group virtually, e.g. via teleconference [73]
The need for the group not to negatively affect members’ emotions or wellbeing, e.g.: - Finding groups anxiety provoking [58, 69] - Feeling uncomfortable being in a group [58] - Being concerned that they are too ‘well’ to attend and may distress others who are experiencing ill health [76]	Acceptability	Implementation/sustainment	X		Opportunities for members to interact in smaller groups beyond the formal group meetings, e.g. out-of-session add-ons/spin-offs, such as lunches, catch-ups, online social media groups [31, 75, 79]
The need for different types of support (e.g. for information about treatment, emotional support, social support) [77, 79] which could be influenced by recency of metastatic diagnosis [79]	Acceptability	Implementation/sustainment	X		
The patients’ need to have someone other than a healthcare professional facilitating the group to allow for honest conversations about experiences of health services [76]	Acceptability	Adoption		X	
Recipients: Opportunity					
Patients’ capacity to attend regularly might be limited by ill health [58]	Feasibility	Adoption/implementation/sustainment	X		Option of joining group virtually, e.g. via teleconference [73] Having flexibility for group to meet outside of usual setting, e.g. person’s home/hospital room [75]
Patients’ capacity to attend might be limited by conflicting commitments (e.g. looking after children, having treatment) [58, 69]	Acceptability	Adoption/implementation/sustainment	X		Option of joining group virtually, e.g. via teleconference[73]
Recipients: Motivation					
Patients are motivated to attend as the group provides an opportunity to give back through knowledge sharing and providing support (reciprocity; mutual aid; meaning making) [79]	Acceptability	Implementation/sustainment		X	
Deliverers: Needs					
Co-facilitation of groups [31, 43]	Feasibility	Sustainment		X	
Deliverers: Capability					
Group facilitators/leaders’ competence in delivery and management of group - Skills, experience and ability to manage difficult situations and conversations [43, 76, 79] - Ability to manage the introduction of new members [58, 79] - Ability to manage the death of member [79]	Acceptability	Implementation/sustainment		X	Appropriately trained staff with relevant expertise to lead a support group [43] Access to training resources/development opportunities [58, 59] - manuals or training workshops [58, 59] - monthly evaluation and review (e.g. review of videos, written feedback [58, 59] - supervision [58, 59] Debriefing and reflection by group facilitators/leaders after each meeting supported development of facilitators’ skills and relevance/acceptability of group to members [43]
Needing a professional to lead group (as patients may need to prioritise their health over the running of the group) [43]	Feasibility	Sustainment	X		
Deliverers: Opportunity					
Group facilitators/leaders’ capacity to: - Follow-up proactively as needed with members outside of sessions [79] - Be accessible and responsive to members between meetings [79] - Meet with potential members prior to joining group to ensure group is a good fit for the person [58, 79]	Acceptability	Implementation/sustainment		X	
Reliance on healthcare professionals to volunteer time to lead group [79]	Feasibility	Sustainability	X		
IMPLEMENTATION PROCESS					
Reflecting and evaluating					
Monthly evaluation and review (e.g. review of videos, written feedback) and supervision [58, 59]	N/A	Implementation		X	Reported above as a possible implementation strategy to improve capability of the support group facilitators to lead/deliver support groups appropriately and effectively

Ten studies (53%) reported barriers to implementation and 9 studies (47%) reported enablers. These barriers and enablers mapped to twelve CFIR constructs across four domains. The CFIR constructs to which most barriers and enablers were coded were as follows: (1) the extent to which the needs of people with advanced or metastatic cancer were accurately known and prioritised by the organisation and staff delivering the support groups (individuals domain/recipients’ needs: 6 different factors mentioned 19 times across 12 articles); (2) the capability of the support group facilitators to deliver the groups (individuals domain/deliverers’ capability: 3 factors mentioned 5 times across 3 studies); and (3) the capacity to adapt the running and delivery of a support group to meet the needs of a particular patient group within a particular organisational setting (innovation domain/adaptability: 2 different factors mentioned 5 times across 3 articles).

Fourteen implementation strategies were identified across eight studies. Five of the strategies supported implementation or sustainment by addressing factors relating to the capability of the people delivering the innovation (i.e. the skills, experience and competence of the support group facilitators). Four of the strategies supported implementation or sustainment by addressing factors relating to the needs of the support group participants. Three of the strategies supported implementation or sustainment by addressing factors relating to the innovation (i.e. the support groups).

By categorising the factors identified as influencing implementation and mapping them to the antecedents of implementation, we are able to demonstrate how the acceptability of an innovation (from the perspective of recipient, i.e. the person affected by cancer, and deliverer, i.e. the support group leader) can potentially impact upon implementation or sustainment (Table 4). For example, even within a group for advanced cancer patients, there was a need for participants to identify with others who shared similar experiences or circumstances such as cancer type, life stage and role (e.g. carer versus patient) [80]. Diversity within the group could affect acceptability and therefore the initial implementation or long-term sustainment of the group. A possible strategy to overcome this challenge of having a diverse group was encouraging opportunities for members to interact in small groups beyond the formal group meetings [31, 75, 79]. Another key factor was the group leaders’ competence in delivery and management of the group, including dealing with difficult conversations, introducing new members and managing deaths of members [43, 58, 76, 79]. Identified strategies included providing appropriate training, access to training resources such as manuals and workshops, monthly reviews and evaluations, debriefing and reflection, and supervision [43, 58, 59].

## Discussion

This scoping review identified 19 articles reporting data on the effectiveness of professionally led support groups for people with advanced or metastatic cancer or on factors influencing their implementation. Notable was that only two studies were published in the past 10 years, both of which were small-scale evaluations of community- or hospital-based groups [79, 80]. All eight RCTs were published between 1981 and 2007 and reported on groups for people with metastatic breast cancer. The relative paucity of relevant recent published research is surprising given the widespread recognition of the benefits of professionally led support groups for people with cancer, the distinctly different clinical and support requirements of people with advanced or metastatic cancers compared with early, potentially curable cancers [7, 8], and the call to prioritise metastatic survivorship research and supportive care [2, 11, 12]. Furthermore, with the emergence in the past 20 years of implementation science as a critical field of study in health services research and psychosocial care in oncology, we had expected more studies to report on determinants, strategies or outcomes relating to implementation [84–86]. Implementation science aims to bridge the gap between what is known (i.e. evidence-based interventions) and what is being done (i.e. policy and practice) [84]. Ultimately, the impact of research innovations on reducing cancer burden and cancer-related health disparities is limited by failures in implementation and scale-up. Our review confirms that many common implementation challenges apply to professionally led advanced or metastatic support groups, including the development of evidence-based innovations that are not necessarily easily implemented in real-world settings, limited planning strategies to enhance delivery of evidence-based innovations and problems adapting existing evidence-based innovations for new settings and populations [84].

Our review identified evidence to support the use of professionally led support groups for people with advanced or metastatic cancer. In particular, RCTs and cohort studies provided evidence for their effectiveness in reducing mood disturbances, distress (traumatic stress and depression) and pain. This is consistent with benefits reported in reviews of the literature, including a meta-analysis of peer support interventions in cancer [87, 88], a review of professionally led cancer support groups [20] and a review of professionally led and peer-led cancer support groups in Australia [89]. Contrary to the literature, benefits for overall quality of life were not observed in the RCTs included in the current review. It is worth noting that most of the existing evidence on quality of life was demonstrated in studies of support groups for early-stage cancer or in studies of groups where stage was not clearly reported [20, 90]. The apparent lack of effectiveness in improving quality of life overall may be due to limitations in the validated scales used to measure quality of life outcomes. Current tools such as the QLQ-C30 lack the ability to capture the impact metastatic breast cancer has on a person’s life. There is currently an urgent need for specific tools to aid in the evaluation of health-related quality of life in metastatic breast cancer [91]. The European Organisation for Research and Treatment of Cancer (EORTC) is currently developing an EORTC module to measure health-related quality of life in people with metastatic breast cancer. The new module will be used in conjunction with EORTC QLQ-C30 and will provide better measurement of the quality-of-life issues experienced by people with metastatic breast cancer. Scales that are commonly used in other health settings might be adapted for this population. For example, the original and abbreviated Duke-UNC Functional Social Support Questionnaire (DUFSS) has been shown to have adequate reliability and validity for measuring perceived social support in the settings of palliative care and oncology [92]. A scale developed specifically to assess existential distress in patients with advanced cancer also showed promising preliminary psychometric properties [93].

Evidence from qualitative and mixed methods studies provided important insights into the psychosocial and informational benefits of attending advanced or metastatic cancer support groups that can be hard to capture using standard quantitative assessment tools [69]. These benefits included a greater sense of social connection and belonging; help dealing with existential distress; access to information and knowledge related to treatment and resources; a greater sense of empowerment and control; improved relationships with family; and help facilitating communication with healthcare professionals. These benefits are consistent with benefits reported in recent reviews of qualitative studies of peer-led cancer support groups (informational support, connection through sharing of experiences) [41] and quantitative studies of peer-support interventions for people with cancer (empowerment, feeling in control) [94]. These benefits parallel outcomes identified as most valued by patients involved in peer support programs in other research [18].

As CFIR 2.0 highlights, successful adoption, implementation and sustainment of an evidence-based innovation require a clear understanding of recipients’ needs [61]. Several of the determinants of implementation success identified in our review related to the particular needs of the innovation recipients, that is, people with advanced or metastatic cancer. Understanding these needs allows for the active adaptation of a support group intervention to a particular setting and patient population [64, 95]. For example, two studies in our review identified that participants’ informational and emotional support needs varied depending on the recency of their metastatic diagnosis, which could be a barrier to the acceptability of the group. The format and content of the support group therefore needed to take into account the needs of people who had recently joined as well as those who had been attending for many years [77, 79]. Acceptability of the group was also affected by the need for group members to travel in order to physically attend the group meetings. Only one study involved teleconference in addition to face-to-face mode of delivery. The increasing use of telehealth services since the COVID-19 pandemic may have removed this barrier for those who cannot attend in person [96]. However, a potential challenge of running groups virtually may be the impact on the group leaders’ capacity to monitor participants’ psychological safety and wellbeing [26]. A further consideration affecting acceptability is that the needs of the group as a whole are likely to change over time. Groups specifically catering for people with advanced or metastatic cancer are inevitably going to experience changes in group membership as members deal with cancer progression, acute periods of illness and eventually death. Several of the studies in our review reported that an enabler of group sustainability was the ability of the group to adapt and evolve in an organic way, for example shifting its psychotherapeutic model (from SEGT to something more akin to mutual aid [97, 98]) and creating a more democratic structure that allowed participants to have a greater say in the running of the group [72, 79].

Just as successful adoption, implementation and sustainment of an evidence-based innovation requires a clear understanding of recipients’ needs, it also requires a clear understanding of the needs of the person delivering the innovation, in this case the support group facilitator [61]. Our review highlighted how the capacity and capability of the facilitators to deliver and appropriately lead the groups was an important enabler. The importance of the support group leaders’ skills and training has been reported for cancer support group leaders in general but not specifically for leaders of advanced or metastatic groups [37, 38, 99]. While Australian research has highlighted the differing experiences and training and support needs of health professionals versus peer leaders [100], we are unaware of any training programs or materials designed specifically for professionals or peers running a support group for people with advanced or metastatic cancer. Important skills identified in this review included the group leaders’ ability to manage difficult conversations within the group and to handle the progression of disease or death of members. While these scenarios are not unique to advanced or metastatic support groups, they are more commonly experienced by this particular population and may require additional training, support or clinical supervision to ensure the wellbeing of participants and facilitators [39].

### Strengths and limitations

A strength of our review is that it focuses specifically on people with advanced or metastatic cancer and on professionally led support groups. Reviews of psychosocial support for people with cancer tend to report effectiveness of peer support programs for cancer patients in general, and rarely provide data specific to those with advanced or metastatic cancer. By including both quantitative and qualitative studies that used a range of study designs, we captured the effectiveness outcomes measured using validated scales (e.g. distress, mood and pain) but also the benefits that have been reported to be valued most by people attending support groups (e.g. reducing isolation, building connection, sharing of experiences). A further strength is that we mapped the implementation antecedents and outcomes to CFIR 2.0, a comprehensive meta-theoretical implementation framework. Mapping to CFIR 2.0 helped us identify and categorise barriers and enablers across different levels, from individuals directly involved in the implementation to the surrounding organisational setting. However, as this mapping was done retrospectively, we cannot be certain that some important domains, constructs, outcomes or antecedents may have been missed.

The review has several limitations. First, much of the evidence for effectiveness comes from the eight RCTs, all of which evaluated the effectiveness of SEGT in patients with metastatic breast cancer. The more recent studies were typically small-scale evaluations of community- or hospital-based groups [79, 80]. In contrast to the earlier RCTs of SEGT, many of these later studies were not implementing a manualised support group intervention; it was therefore not always clear what the components or the ‘active ingredients’ of the intervention were [101]. Furthermore, the inconsistency of measures and follow-up intervals made it difficult to compare effectiveness outcomes across studies. Most trial studies assessed benefits of the group for patients with metastatic breast cancer 1 year after joining. In the absence of ongoing, regular evaluations, it is unclear if these benefits were sustained beyond the follow-up periods. Second, clear patterns of implementation outcomes could not be observed due to the lack of standardised measures as well as inconsistency in the reporting of implementation results. Some of the data on implementation barriers and facilitators were reported anecdotally by the study authors when discussing the effectiveness outcomes of the support group or describing the process of setting up the group. Thus, the quality of the data relevant to the implementation outcomes was inconsistent across the studies. A similar limitation was reported in a recent review of cancer peer support interventions for people with advanced cancer [102]. As Walshe and colleagues pointed out, non-standardised reporting has implications for both implementation practice and study replication. The recent focus on more transparent reporting of trials including guidelines for reporting implementation outcomes means that future studies are more likely to report the data critical to implementation and replication [103–105]. Finally, there was limited evidence identified in the review on the benefits of support groups for families and carers of people with advanced or metastatic cancer. Given the growing population of people living with cancer, more attention needs to be paid to families and carers and their unmet needs for psychosocial support.

## Conclusion

In line with the literature on the effectiveness of cancer support groups in general, this review found evidence of the effectiveness of professionally led support groups for people with advanced or metastatic cancer. However, of the 19 studies, only two were published in past 10 years. All eight of the RCTs were undertaken 20 years ago in women with metastatic breast cancer. While studies in women with metastatic breast cancer are important, improvements in cancer treatments have resulted in a growing population of people living long-term with other types of advanced or metastatic cancer. Research in other cancers is required to ensure the suitability of support groups to the target audience. In relation to the mode and format of the group, the evidence identified comes primarily from high-intensity face-to-face programs. One of the biggest issues in Australia and internationally is that of the psycho-oncology workforce. Many of these groups reported in this review were relatively time and labour intensive, some meeting weekly for more than 2 h. Running such groups in this format might be challenging even in large comprehensive, city-based services, much less in regional areas. In a post-pandemic era where telehealth is far more widely available, it is clear that further research is needed to understand what contemporary support groups for people with advanced or metastatic cancer look like, their benefits and factors that hinder or support their set-up and long-term sustainability. It will also be crucial to gain a clearer understanding of the necessary training and capabilities required for leaders of advanced or metastatic support groups, so that they can effectively lead these groups.

## Supplementary information

ESM 1 (PDF 111 kb)
