# Supplementary material for: Professionally led support groups for people living with advanced or metastatic cancer: a systematic scoping review of effectiveness and factors critical to implementation success within real-world healthcare and community settings
Source: J Cancer Surviv. 2024 Jan 8;19(3):957–77. doi: 10.1007/s11764-023-01515-w (PMC12081543; doi:10.1007/s11764-023-01515-w)
Supplement: Supplementary file 1 — (PDF 111 kb) [file 11764_2023_1515_MOESM1_ESM.pdf]

Key concepts and Mesh terms

KEY CONCEPTS		
Cancer	Advanced stage	Support groups
**KEYWORDS**		
Cancer	Advance	"Support group"
Neoplasm	Metastasis	"Support intervention"
Tumor or tumour*	Disseminated	Self-help group*
Oncology	Progression	Online support group*
	Inoperable	Social media support group*
	Incurable	
	Chronic	
	Recurrent	
	Relapse	
	Palliative	
	Protracted	
	"end stage"	
**MeSH Subject Headings**		
exp Neoplasm/		Social support/
exp Cancer/		Psychosocial support systems/
Neoplasm Metastasis/		
Oncology/		
Psycho-Oncology/		

Search string MEDLINE and PsychINFO (15 December 2021)

# ▲	Searches
1	cancer.mp.
2	exp Cancer/
3	((tumour* or tumor*) adj3 cancer*).tw.
4	oncolog*.mp. or Integrative Oncology/ or Psycho-Oncology/
5	exp Neoplasms/
6	Neoplasm Metastasis/
7	or/1-6
8	Disease progression/
9	(and/7) adj3 (advance* or metasta* or dissemin* or progress* or incurab* or inoperab* or chronic or recurr* or relaps* or palliative* or protracted or "end stage").mp.
10	or/8-9
11	social support/ or psychosocial support systems/
12	("support group" or "support intervention").mp. [mp=title, abstract, heading word, table of contents, key concepts, original title, tests & measures, mesh word]
13	Self-Help Groups/
14	Self-help group*.mp.
15	Support group*.mp.
16	(Online support group* or social media support group*).mp. [mp=title, abstract, heading word, table of contents, key concepts, original title, tests & measures, mesh word]
17	or/11-16
18	and/7,10,17

Search string CINAHL (15 December 2021)

☐	S8	S1 AND S6 AND S7	**Expanders** - Apply equivalent subjects<br>**Search modes** - Boolean/Phrase
☐	S7	S2 OR S3	**Expanders** - Apply equivalent subjects<br>**Search modes** - Boolean/Phrase
☐	S6	S4 OR S5	**Expanders** - Apply equivalent subjects<br>**Search modes** - Boolean/Phrase
☐	S5	"support groups or support intervention or psychosocial support"	**Expanders** - Apply equivalent subjects<br>**Search modes** - Boolean/Phrase
☐	S4	(MM "Support Groups+") OR (MM "Psychotherapy, Group+") OR (MM "Support, Psychosocial+")	**Expanders** - Apply equivalent subjects<br>**Search modes** - Boolean/Phrase
☐	S3	"(advance* OR mestasta* OR dissemin* OR progress* OR incurab* OR inoperab* OR chronic OR recurr* OR relaps* OR palliative OR protracted OR "end stage")" OR (MH "Neoplasm Metastasis+") OR "metastasis" OR (MH "Palliative Care") OR "palliative" OR "end stage"	**Expanders** - Apply equivalent subjects<br>**Search modes** - Boolean/Phrase
☐	S2	(MM "Disease Progression+") AND "disease progression"	**Expanders** - Apply equivalent subjects<br>**Search modes** - Boolean/Phrase
☐	S1	"cancer" OR (MM "Neoplasm Metastasis+") OR "tumour*" OR (MM "Oncology+") OR "oncology" OR (MH "Psycho-Oncology") OR (MH "Oncologic Care+")	**Expanders** - Apply equivalent subjects<br>**Search modes** - Boolean/Phrase
